# The BCR-ABL1 Inhibitors Imatinib and Ponatinib Decrease Plasma Cholesterol and Atherosclerosis, and Nilotinib and Ponatinib Activate Coagulation in a Translational Mouse Model

**DOI:** 10.3389/fcvm.2018.00055

**Published:** 2018-06-12

**Authors:** Marianne G. Pouwer, Elsbet J. Pieterman, Lars Verschuren, Martien P. M. Caspers, Cornelis Kluft, Ricardo A. Garcia, Jurjan Aman, J. Wouter Jukema, Hans M. G. Princen

**Affiliations:** ^1^Metabolic Health Research, Gaubius Laboratory, The Netherlands Organization of Applied Scientific Research (TNO), Leiden, Netherlands; ^2^Department of Cardiology, Leiden University Medical Center, Leiden, Netherlands; ^3^Microbiology and Systems Biology, The Netherlands Organization of Applied Scientific Research (TNO), Zeist, Netherlands; ^4^Good Biomarker Sciences, Leiden, Netherlands; ^5^Cardiovascular Drug Discovery, Bristol-Meyers Squibb, New York, United States; ^6^Departments of Physiology and Pulmonary Diseases, VU University Medical Center, Amsterdam, Netherlands

**Keywords:** atherosclerosis, animal model cardiovascular disease, lipids and lipoprotein metabolism, coagulation/thrombosis, cholesterol, chronic myeloid leukemia, cardiovascular side effects

## Abstract

Treatment with the second and third generation BCR-ABL1 tyrosine kinase inhibitors (TKIs) increases cardiovascular risk in chronic myeloid leukemia (CML) patients. We investigated the vascular adverse effects of three generations of TKIs in a translational model for atherosclerosis, the APOE*3Leiden.CETP mouse. Mice were treated for sixteen weeks with imatinib (150 mg/kg BID), nilotinib (10 and 30 mg/kg QD) or ponatinib (3 and 10 mg/kg QD), giving similar drug exposures as in CML-patients. Cardiovascular risk factors were analyzed longitudinally, and histopathological analysis of atherosclerosis and transcriptome analysis of the liver was performed. Imatinib and ponatinib decreased plasma cholesterol (imatinib, −69%, *p* < 0.001; ponatinib 3 mg/kg, −37%, *p* < 0.001; ponatinib 10 mg/kg−44%, *p* < 0.001) and atherosclerotic lesion area (imatinib, −78%, *p* < 0.001; ponatinib 3 mg/kg, −52%, *p* = 0.002; ponatinib 10 mg/kg, −48%, *p* = 0.006), which were not affected by nilotinib. In addition, imatinib increased plaque stability. Gene expression and pathway analysis demonstrated that ponatinib enhanced the mRNA expression of coagulation factors of both the contact activation (intrinsic) and tissue factor (extrinsic) pathways. In line with this, ponatinib enhanced plasma levels of FVII, whereas nilotinib increased plasma FVIIa activity. While imatinib showed a beneficial cardiovascular risk profile, nilotinib and ponatinib increased the cardiovascular risk through induction of a pro-thrombotic state.

## Introduction

Chronic myeloid leukemia (CML) is a myeloproliferative neoplasm caused by a translocation of the chromosomes 9 and 22 that results in formation of the *Bcr-Abl1* oncogene (1) and a constitutively active c-Abl kinase domain, which drives uncontrolled cell growth and tumorigenesis.

Patients with CML are treated with specific tyrosine kinase inhibitors (TKIs). The first-line TKI imatinib is widely used and has proven to be successful in the treatment of CML. However, relapses are seen in up to 17% of patients treated with imatinib (2) due to amplification and mutations in the *Bcr-Abl1* gene (3) that lead to imatinib resistance. The second and third generation TKIs, nilotinib and ponatinib among others, are effective against these mutations (3), and promising results have been found in relapsed patients (4). Unfortunately, side effects have been reported in patients receiving these TKIs including myocardial infarction and progressive arterial occlusive disease (PAOD) (5–7). As a result, ponatinib was temporarily removed from the US market, and was later reintroduced for the treatment of patients with T315I-positive CML or those in whom no other TKI was indicated.

Since the first reports of vascular adverse effects (VAEs), many authors related the adverse effects of TKI treatment to atherosclerosis and abnormal platelet function (4, 7–9). However, it is still unclear whether the side effects are caused by enhanced vascular inflammation and endothelial dysfunction, atherosclerosis development, increased thrombotic activity *per se*, or a combination of these processes. Furthermore, the underlying disease has been reported to affect metabolic parameters (10) and coagulation (11), which may interfere with the onset of the side-effects upon treatment. Therefore, to elucidate the role of TKI treatment on VAE’s independently of a background of leukemia, we performed a detailed experimental study in healthy pro-atherogenic mice. The aim of this study was to assess the (cardio)vascular side effects of the second and third generation of TKIs, nilotinib and ponatinib, and to compare their effects to the first generation TKI imatinib.

In this study, we used the APOE*3Leiden.CETP mouse as a well-established model for dyslipidemia and atherosclerosis, with a human-like lipoprotein metabolism and atherosclerosis development (See Supplemental material online for more detailed information on the background of the APOE*3Leiden.CETP mice and their response to hypolipidemic drugs; Data Sheet S1). These mice show a human-like response to all lipid-modulating interventions that are being used in the clinic (12–18) and have been used previously to investigate the underlying mechanism of cardiovascular safety issues (19).

We found that imatinib and ponatinib decreased plasma cholesterol, which was associated with decreased atherosclerosis development. Gene expression and pathway analysis demonstrated adverse alterations in genes involved in coagulation which were in line with increased plasma levels of FVII and FVIIa by ponatinib and nilotinib respectively, pointing towards thrombosis instead of atherosclerosis as inducer of the VAEs.

## Materials and Methods

### Animals

Female APOE*3Leiden.CETP transgenic mice (9 to 14 weeks of age) from the SPF breeding stock at TNO-Metabolic Health Research (TNO-Leiden) were used in this study. Females were used because they are more responsive to dietary cholesterol and fat than males. APOE*3Leiden females have a higher VLDL production (20) than males resulting in higher plasma total cholesterol (TC) and triglyceride (TG) levels and more pronounced development of atherosclerosis (21, 22). During the study, mice were housed under standard conditions with a 12 h light-dark cycle and had free access to food and water. Body weight, food intake and clinical signs of behavior were monitored regularly during the study. Animal experiments were approved by the Institutional Animal Care and Use Committee of The Netherlands Organization for Applied Research under registration number 3557.

### Experimental Design and Analyses

Mice were fed a semi-synthetic diet, containing saturated fat from 15% (w/w) cacao butter and 0.15% cholesterol (Western-type diet [WTD]; Hope Farms, Woerden, The Netherlands). All studies started after a run-in period of 3 weeks on WTD, which is designated as T = 0 weeks/baseline, after which mice were matched into groups based on body weight, total cholesterol, plasma triglycerides and age. For the pharmacokinetic (PK) study, mice were randomized in 3 groups (*n* = 9 per group) and received a single oral gavage with imatinib (100 mg/kg), nilotinib (50 mg/kg) or ponatinib (5 mg/kg). At 0.5, 1 and 2 h after oral gavage, blood was sampled from 3 mice per group per time point, and at 4, 7 and 24 h blood was collected by heart puncture after sacrifice. For the (cardio)vascular risk factor andatherosclerosis study, mice were randomized in 6 groups (*n* = 15 per group, *n* = 20 in control group) and received, based on the results of the PK study, a once-daily oral gavage with nilotinib (10 or 30 mg/kg), ponatinib (3 or 10 mg/kg), or a twice-daily gavage with imatinib (150 mg/kg). The TKIs were suspended in 5% carboxymethyl cellulose and all mice except the imatinib group received a second oral gavage with the vehicle alone (5% carboxymethyl cellulose). The TKIs were purchased at LC laboratories, Woburn (MA), USA. After 12 weeks 5 mice of the control group were sacrificed to asses atherosclerosis development and to determine the end-point of the study. After sixteen weeks of treatment all animals were sacrificed by CO_2_ inhalation. Plasma cholesterol, triglycerides, HDL-C, lipoprotein profiles, SAA, E-selectin and MCP-1, aspartate transaminase (AST) and alanine transaminase (ALT) were measured throughout the study. Blood pressure was measured at 2 and 15 weeks of treatment. Measurement of hepatic lipid and protein content; protein and albumin content in broncho-alveolar lavage (BAL) fluid; urinary albumin/creatinine levels; and histology of lung and hearts was performed at 16 weeks. Total FVII coagulant activity was measured at 4 and 12 weeks and FVIIa activity at 4 weeks. Gene expression analysis using Next Generation Sequencing with the Illumina Nextseq 500 and subsequent pathway analysis of liver of 8 mice per group was performed following established protocols (23, 24).

### Statistical Analysis

Significance of differences between the groups was calculated in SPSS 22.0 for Windows. Normally distributed data was tested parametrically using a one-way ANOVA for multiple comparisons with a Dunnett’s post-hoc test. Non-parametric data were compared separately with a Mann-Whitney U test with adjusted rejection criteria using a Bonferroni-Holm correction. Correlations between lesion size (after square root transformation) and cholesterol exposure were calculated with a Pearson’s correlation test. All groups were compared with the control group. Values are presented as means ± SD and *p*-values < 0.05 were considered statistically significant.

For a more detailed description of the applied methods, please see the Supplementary material online (Data Sheet S1).

## Results

### PK Analysis and Plasma Drug Concentrations

Pharmacokinetic analysis was performed after a single dose of imatinib (100 mg/kg), nilotinib (50 mg/kg) or ponatinib (5 mg/kg) (Table 1) and based on these results the doses for the atherosclerosis study were adjusted to twice daily 150 mg/kg for imatinib, once daily 10 or 30 mg/kg for nilotinib and once daily 3 or 10 mg/kg for ponatinib. The relatively high dose of imatinib needed to achieve plasma concentrations comparable to those in CML-patients was due to the short half-life of the drug in mice and is in line with previous reports (29). Drug exposure after repeated dosing was measured in sacrifice plasma and calculated AUCs were similar to those in CML-patients for imatinib and the low doses of nilotinib and ponatinib (Table 1).

**Table 1:** Pharmacokinetics of imatinib, nilotinib and ponatinib in APOE*3Leiden.CETP mice and CML-patients.

**TKI**	APOE*3Leiden.CETP mice					**CML patients**				
	**Dose (mg/kg)**		**T_max _(h)**	**C_max_ (µg/mL)**	AUC_0–24_ (µg/mL*h)	**Dose (mg/kg)**		**Subject**	AUC_0–24_ (µg/mL*h)	**Reference**
**Imatinib**	Single	100	2.28	5.29	32.79	Day 1, BID	400	CML-patients	36.2 ± 7.4	(25)
	Repeated dose, BID	150	2.47	11.07	78.13	Steady state, BID	400	CML-patients	68.4 ± 29.8	(25)
**Nilotinib**	Single	50	3.26	10.97	117.49	Single, QD	400	Healthy subjects	14.7 ± 5.0	(26)
	Repeated dose, QD	10	3.17	2.30	24.60	Repeated dose, QD	400–1,200	CML-patients	36.0 ± 11.8	(26)
	Repeated dose, QD	30	3.17	6.89	73.81					
**Ponatinib**	Single	5	8.00	0.19	3.24	Single, QD	60	CML-patients	0.7 ± 0.4	(27)
	Repeated dose, QD	3	6.72	0.14	2.43	Repeated dose, QD	15–60	CML-patients	1.3	(28)
	Repeated dose, QD	10	6.73	0.46	8.11					

T_max_, C_max_ and AUC_0–24_ were calculated after a single dose or after 16 weeks of treatment (repeated dose) for imatinib, nilotinib and ponatinib and compared with plasma concentrations in CML patients. AUC, area under the curve; BID, twice a day; CML, chronic myoloid leukemia; QD, once a day. Data are presented as means (APOE*3Leiden.CETP mice) and means ± SD (CML-patients), unless SD was not given in the reports.

### Safety Aspects of Treatments with TKIs

No clinical signs of deviant behavior and no effects on body weight and food intake were noted in any treatment group as compared with control. Plasma ALT and AST, measured at the start and after 16 weeks of treatment, showed no aberrant results (Table S1; Data Sheet S1). The number of circulating peripheral blood mononuclear cells (PBMC’s) in the blood as measured by fluorescence-activated cell sorting (FACS) analysis at 12 weeks (Table S2; Data Sheet S1) was reduced by imatinib (−42%, *p* = 0.006) and by the high-dose ponatinib (−44%, *p* = 0.003). In addition, imatinib and the high doses of nilotinib and ponatinib decreased pro-inflammatory Ly6C^high^ monocytes, all consistent with the mode of action of TKIs. Two mice (ponatinib 3 mg/kg) died during blood pressure measurements at t = 15 weeks, and one mouse (nilotinib 30 mg/kg) was excluded from atherosclerosis measurement due to deviating heart anatomy.

### (Cardio) Vascular Risk Factors

#### Imatinib and Ponatinib Reduce Plasma Cholesterol Levels

**Figure 1:**
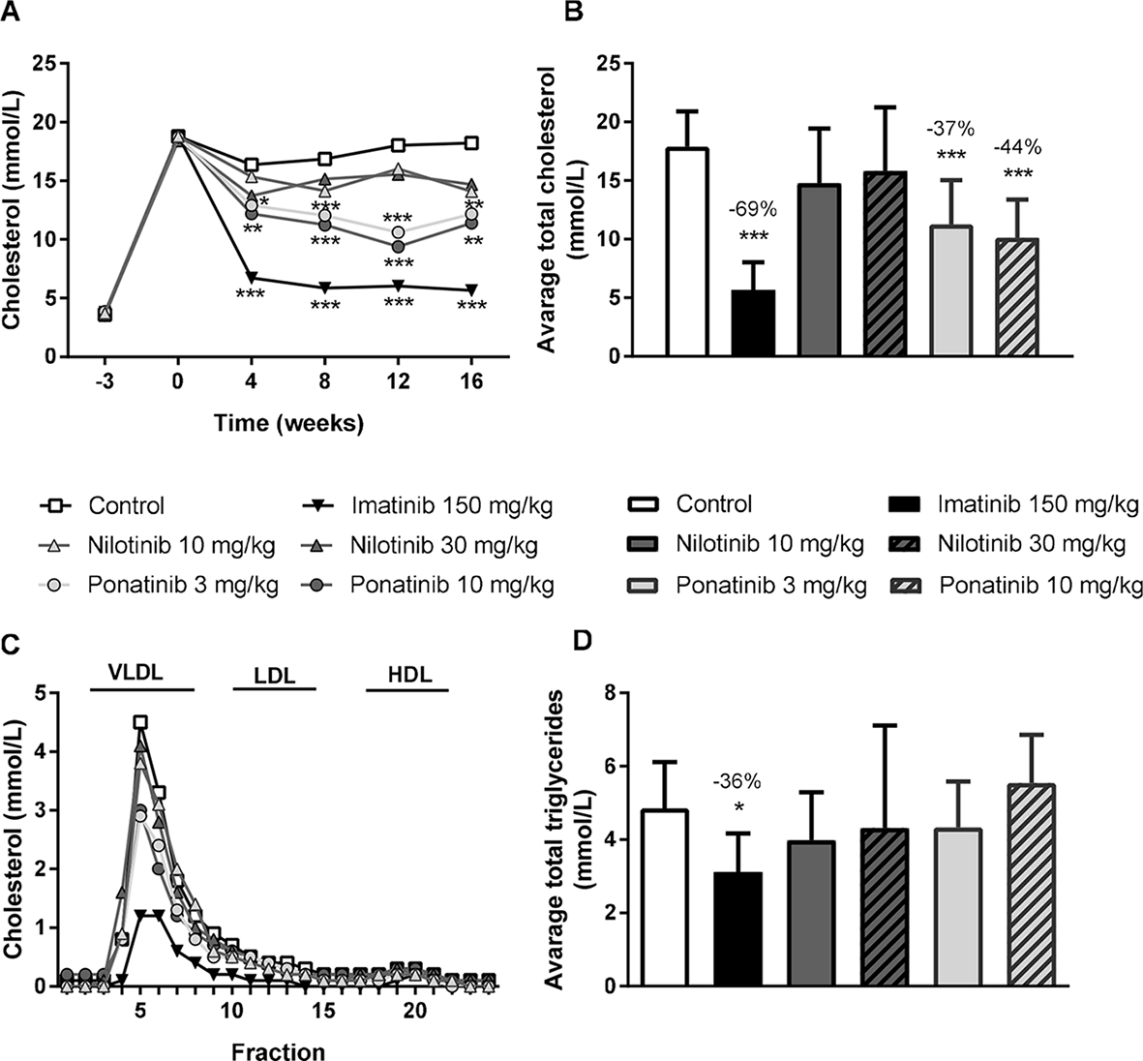
Imatinib and ponatinib reduce plasma cholesterol levels and imatinib decreases average triglycerides. Plasma cholesterol was measured throughout the 16 week study **(A)** and average plasma cholesterol **(B)** and triglycerides **(D)** were calculated. Lipoprotein profiles were assessed by FPLC lipoprotein separation after 16 weeks of treatment **(C).** FPLC, Fast protein liquid chromatography; VLDL, very-low-density-lipoprotein; LDL, low-density-lipoprotein; HDL, high-density-lipoprotein. *p < 0.05, **p < 0.01 ***p < 0.001. Data are presented as means ± SD (*n* = 13–15 per group).

As dyslipidemia is a major risk factor for cardiovascular disease, we measured plasma cholesterol and triglyceride levels throughout the study, and HDL-C at the end point. The Western-type diet resulted in an average plasma cholesterol of 17.3 ± 3.5 mmol/L, triglycerides of 3.3 ± 1.0 mmol/L, and an HDL-C level of 1.4 ± 0.2 mmol/L in the control group. When compared to the control group, imatinib reduced average cholesterol and triglyceride levels by 69% (*p* < 0.001) and 36% (*p* = 0.019), respectively. Ponatinib decreased cholesterol levels by 37% (3 mg/kg, *p* < 0.001) and 44% (10 mg/kg, *p* < 0.001), whereas nilotinib had no significant effect on plasma lipid levels (Figure 1A–D). At 16 weeks of treatment, HDL-C was decreased by ponatinib in both the low (−30%, *p* = 0.003) and high (−25%, *p* = 0.016) dose. The reduction of plasma cholesterol by imatinib and ponatinib was mainly confined to VLDL-LDL (i.e., non-HDL) (Figure 1C).

These findings show that imatinib decreases plasma cholesterol and triglyceride levels in APOE*3Leiden.CETP mice, which is consistent with the cholesterol-lowering effect observed in patients (30–33).

#### Ponatinib and Imatinib Decrease Hepatic Lipid Content

**Figure 2:**
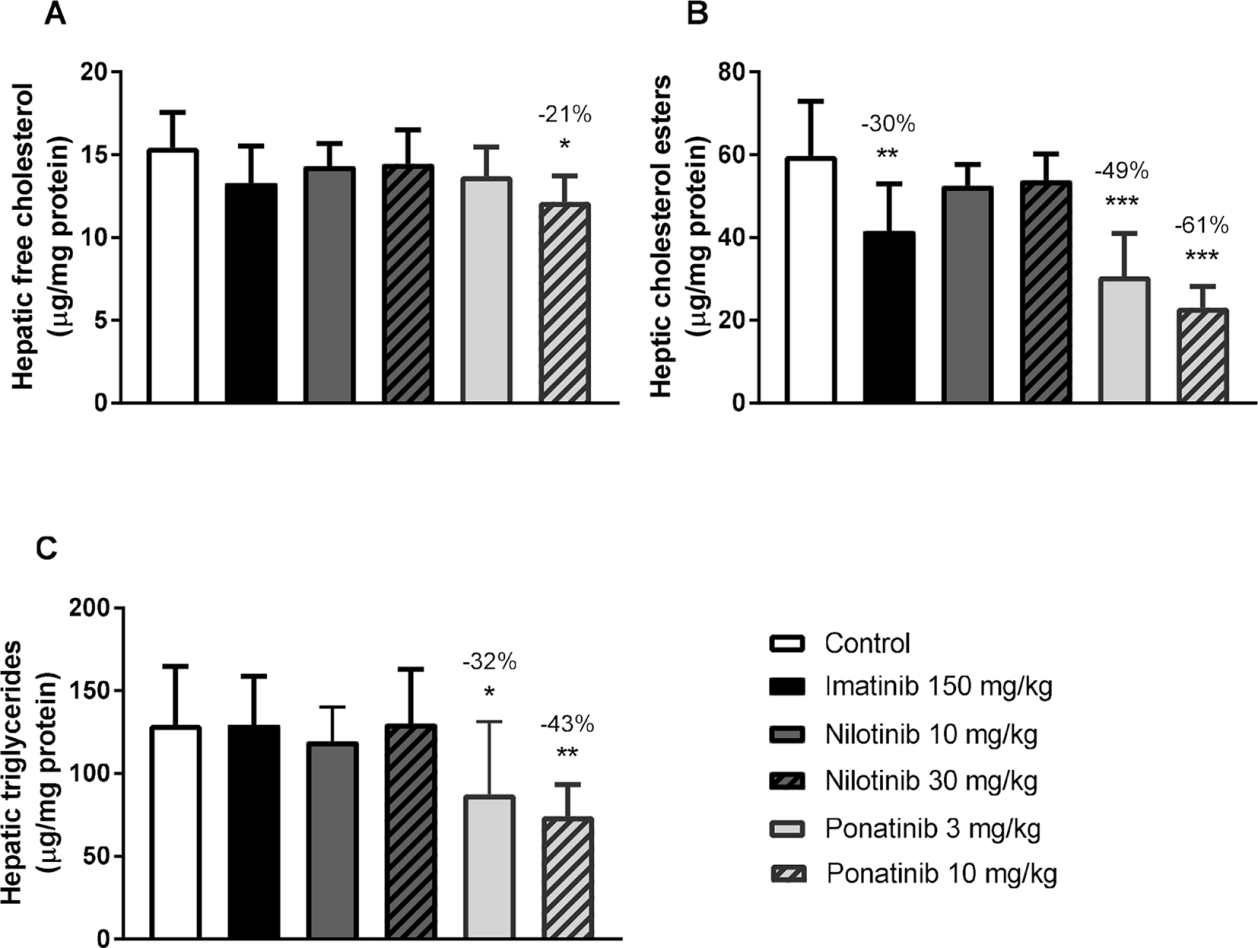
Imatinib and ponatinib decrease hepatic lipid content. Hepatic free cholesterol **(A)**, cholesterol ester **(B)** and triglyceride **(C)** content were measured by HPTLC after 16 weeks of treatment. HPTLC, high-performance thin-layer chromatography. *p < 0.05, **p < 0.01 ***p < 0.001. Data are presented as means ± SD (*n* = 8 per group).

Liver lipid storage may give insight into how lipid metabolism is affected by imatinib and ponatinib. Therefore, hepatic lipid content was measured by HPTLC. Free cholesterol content was decreased by 10 mg/kg ponatinib (−21%, *p* = 0.007) (Figure 2A), cholesterol ester content was decreased by imatinib (−30%, *p* = 0.001) and by both the low (−49%, *p* < 0.001) and high (−61%, *p* < 0.001) dose of ponatinib (Figure 2B). Triglyceride content was decreased by the low (−32%, *p* = 0.048) and high (−43%, *p* = 0.005) dose of ponatinib (Figure 2C). These data point to a shortage of cholesterol in the liver and suggest that not cholesterol clearance, but VLDL production and/or intestinal absorption of cholesterol are affected by ponatinib and imatinib.

#### Blood Pressure and Vascular Dysfunction

Increased blood pressure and endothelial activation may lead to vascular dysfunction and atherosclerosis. Systolic blood pressure (SBP), measured after 2 and 15 weeks of treatment, was 91 ± 7 and 86 ± 5 mmHg, respectively, in the control group, and heart rate was 726 ± 46 and 716 ± 29 beats per minute. SBP and heart rate were not affected by imatinib, nilotinib or ponatinib treatment. As markers of vascular integrity and leakage or edema formation, we evaluated lung histology, determined the wet/dry weight ratio of the lungs and measured the amount of protein in the BAL fluid. None of the anti-CML drugs showed significant effects on histology and wet/dry weight ratio (data not shown), and ponatinib decreased the amount of protein in BAL fluid by 51% (3 mg/kg, *p* = 0.029) and by 47% (10 mg/kg, *p* = 0.041) (Figure S1A; Data Sheet S1). In contrast, ponatinib increased the urinary albumin/creatinine ratio (approximately 13-fold, N.S.), mainly due to 3 mice with very high levels of urinary albumin (Figure S1B; Data Sheet S1).

These data indicate that the anti-CML drugs did not cause damage to the microvasculature in the lungs but that ponatinib may lead to microvascular dysfunction in the kidney.

#### Ponatinib Increases Plasma E-Selectin

Inflammation is widely considered as an important contributing factor to cardiovascular events (34). Therefore, we measured plasma levels of macrophage-derived chemokine monocyte chemoattractant protein-1 (MCP-1), the adhesion molecule E-selectin as marker of endothelial activation, and serum amyloid A (SAA), an acute phase protein mainly produced by the liver (Table 2). None of the inflammatory markers were significantly altered by imatinib. The Western-type diet increased MCP-1 relative to baseline (T = 0 weeks) (+126%, *p* = 0.015), as did nilotinib (10 mg/kg, +220%, *p* < 0.001; 30 mg/kg, +160%, *p* < 0.001). Ponatinib increased SAA relative to baseline (+123%, *p* = 0.013), but not relative to control. In four of the fifteen ponatinib-treated mice, E-selectin levels were 3 to 10 times increased, leading to an overall increase of 161% (*p* < 0.001) when compared to control, which may point to endothelial activation by ponatinib. Collectively, these data confirm the safety profile of imatinib and suggest endothelial activation and potential endothelial dysfunction in some animals by ponatinib .

**Table 2:** Ponatinib increases markers of inflammation.

**Treatment**	**Dose (***mg/kg)*	**MCP-1 ***(pg/mL)*	**SAA (***µg/mL)*	**E-selectin*** (ng/mL)*
Baseline	-	45 ± 23	6.8 ± 4.4	87 ± 9
Control	-	102 ± 44^†^	10.1 ± 1.2	96 ± 18
Imatinib	150	54 ± 44	9.6 ± 0.5	71 ± 18
Nilotinib	10	144 ± 82^‡^	10.6 ± 1.0	79 ± 23
	30	117 ± 60^‡^	10.6 ± 1.1	99 ± 17
Ponatinib	3	78 ± 29	10.4 ± 1.9	77 ± 22
	10	92 ± 68	15.2 ± 21.1^†^	250 ± 307^‡/*^

Effect of imatinib, nilotinib and ponatinib on inflammatory markers as measured at baseline (T = 0 weeks) and after 16 weeks of treatment. MCP-1, monocyte chemoattractant protein-1; SAA, serum amyloid A..

* p < 0.001, as compared to control.

† p < 0.05. as compared to baseline.

‡ p < 0.001 as compared to baseline. Data are presented as means ± SD (n = 8–15 per group).

### Atherosclerosis

#### Imatinib and Ponatinib Reduce Lesion Size and Severity

**Figure 3:**
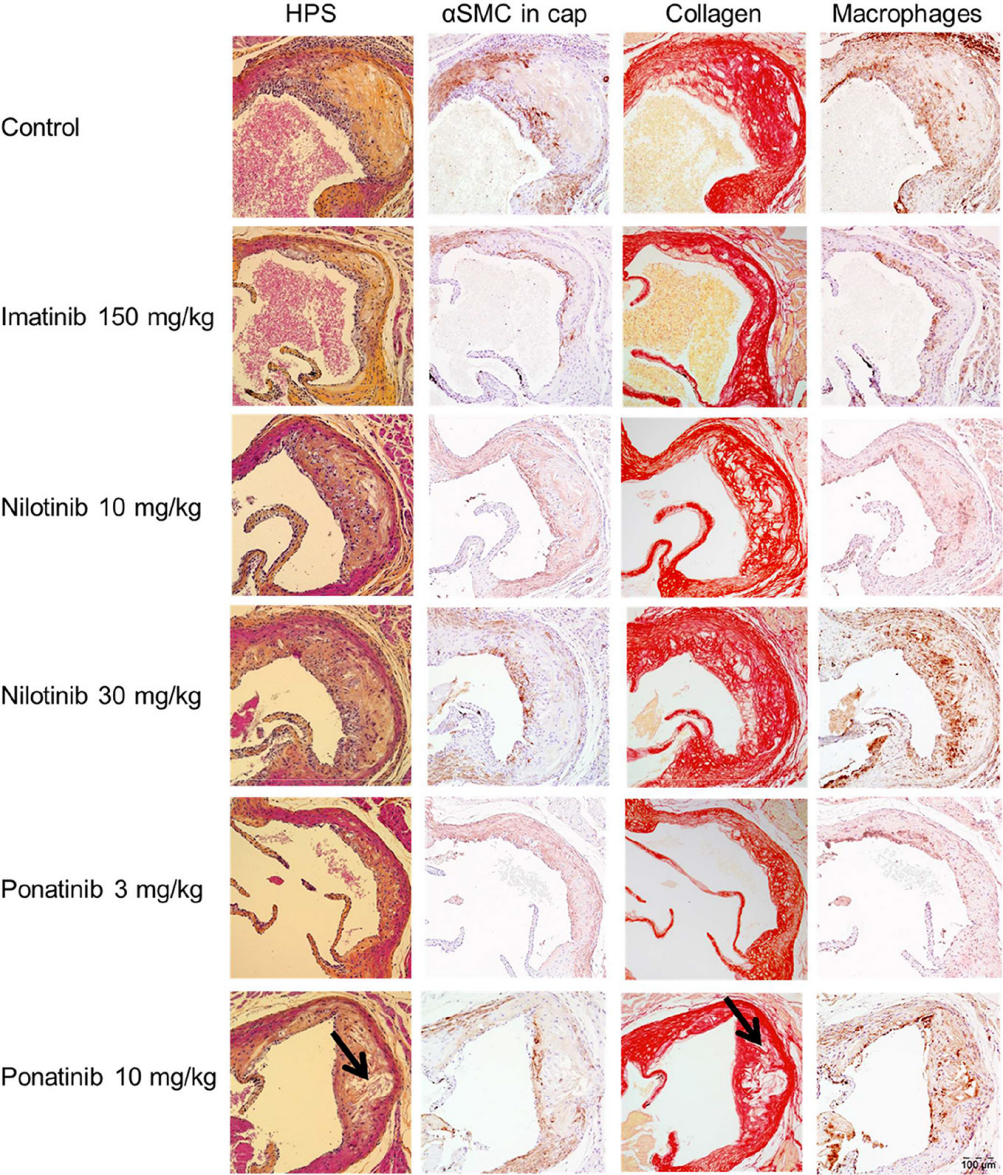
Effect of imatinib, nilotinib and ponatinib on plaque composition. Representative images of HPS staining, immunostaining with α-actin for SMCs, Sirius red staining for collagen and immunostaining with Mac-3 for macrophages. The arrows depict necrotic areas, including cholesterol clefts. Images were taken with an Olympus BX 51 microscope using Olympus analySIS image processing software cell^D. Original magnification ×16. HPS, hematoxylin-phloxine-saffron; SMCs, smooth muscle cells; MAC-3, Purified anti-mouse CD107b Mac-3 Antibody.

**Figure 4:**
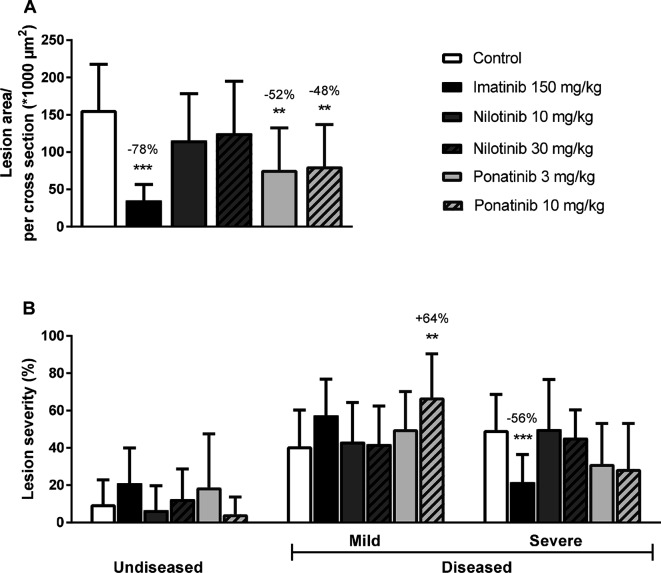
Imatinib and ponatinib reduce atherosclerotic progression. After 16 weeks of treatment, the total lesion area per cross-section was assessed **(A)**. Lesion severity was assessed, categorized as no lesions/undiseased, mild lesions (type I-III) and severe lesions (type IV-V) and expressed as percentage of total lesion area. **(B)**. **p < 0.01 ***p < 0.001. Data are presented as means ± SD (*n* = 13–15 per group).

**Figure 5:**
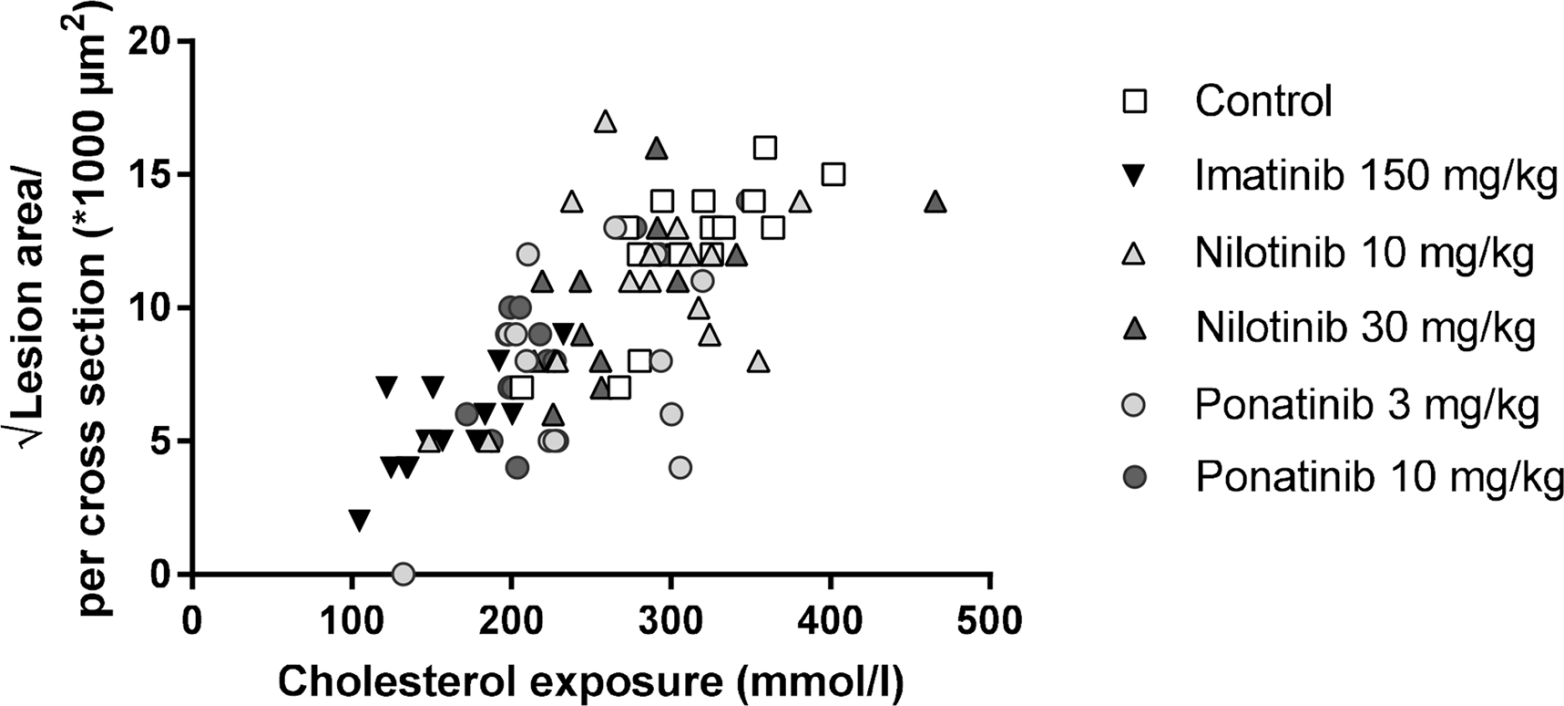
Atherosclerotic lesion area is correlated with cholesterol exposure. Correlation between cholesterol exposure (mmol/L*weeks) and the square root of the lesion area was calculated with a Pearsons’s correlation test (*n* = 13–15 per group).

Next we analyzed the effects of long-term exposure of the anti-CML TKIs on the progression of atherosclerosis as a cardiovascular endpoint, as shown by representative images (Figure 3**)**. Sixteen weeks of Western diet resulted in the development of 4.0 ± 0.8 atherosclerotic lesions and 156 ± 61*1,000 µm^2^ lesion area per cross-section in the control group (Figure 4A). Approximately 55% of these lesions were severe (type IV–V) lesions and only 10% of the segments were unaffected (Figure 4B). The total number of lesions was not affected by any treatment (data not shown), but imatinib and ponatinib diminished the lesion area by 78 (*p* < 0.001), 52 (3 mg/kg, *p* = 0.002), and 48% (10 mg/kg, *p* = 0.006), respectively (Figure 4A). In addition, the total lesion area that consisted of severe lesions was reduced by imatinib (−56%, *p* < 0.001) (Figure 4B). Next, we evaluated whether this anti-atherogenic effect of imatinib and ponatinib could be explained by the reduction in plasma cholesterol levels. The square root of the lesion size was positively correlated with plasma cholesterol exposure (mmol/L*weeks) in control, imatinib and high dose nilotinib and ponatinib treated mice (control R^2^ = 0.79, *p* = 0.001; imatinib R^2^ = 0.71, *p* = 0.003; ponatinib 10 mg/kg R^2^ = 0.79, *p* < 0.001; nilotinib 30 mg/kg R^2^ = 0.63, *p* = 0.016) (Figure 5). Lesion size per cross-section was not correlated with inflammation markers or blood pressure, indicating a dominant role of plasma cholesterol and cholesterol-lowering by the drugs in the development of atherosclerosis.

#### Imatinib Improves Plaque Morphology

**Figure 6:**
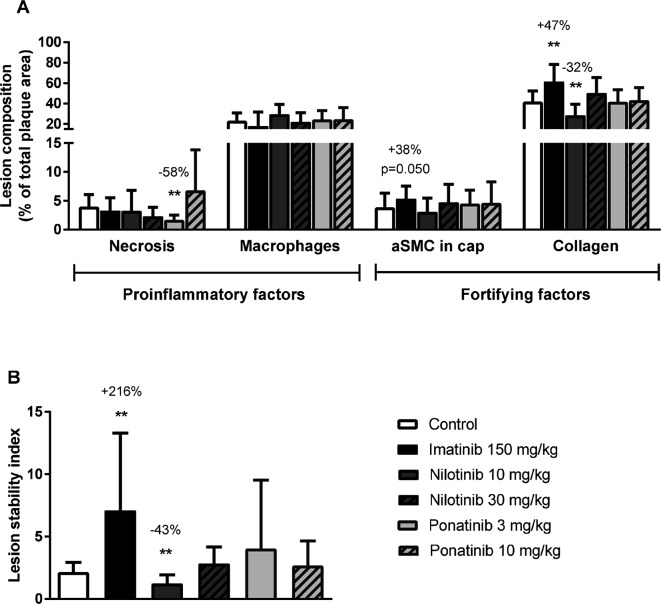
Imatinib increases plaque stability. Necrotic and macrophage content as pro-inflammatory factors, and αSMCs and collagen as fortifying factors, were determined in the severe (type IV-V) lesions and expressed as percentage of total plaque area **(A)**. Plaque stability index was calculated **(B)**. aSMC; α smooth muscle cell., **p < 0.01. Data are presented as means ± SD (*n* = 13–15 per group).

To assess the plaque phenotype as a marker for vulnerability to rupture, lesion morphology was analyzed in the type IV and V lesions from mice treated with imatinib and the high dosages of nilotinib and ponatinib, as shown by representative images (Figure 3**)**. The macrophage and necrosis content were quantified as factors that decrease plaque stability, and smooth muscle cell area in the cap of the lesions and collagen as factors that improve plaque stability (17, 35, 36) (Figure 6A). Average macrophage, necrotic core, collagen and smooth muscle cell content in the control group were 24 ± 8%, 4 ± 2%, 42 ± 10% and 4 ± 3%, respectively (Figure 6A). Imatinib increased the collagen content by 47% (62 ± 17%, *p* = 0.004) and tended to increase αSMC content (+38%, *p* = 0.050), resulting in an enhanced lesion stability index (+216%, *p* = 0.004) (Figure 6B). Nilotinib (10 mg/kg) decreased collagen content (−32%, *p* = 0.003), resulting in a decreased lesion stability index (−43%, *p* = 0.003). Ponatinib (3 mg/kg) decreased necrotic core content (−58%, *p* = 0.001) without affecting plaque stability. Collectively, these data indicate that imatinib induces a more stable plaque phenotype with collagen-rich lesions.

### Transcriptome Analysis

#### Ponatinib Adversely Alters Gene Expression of Coagulation Factors

To find early molecular signatures of other clinically relevant processes induced by the anti-CML drugs, gene expression and pathway analysis was performed in the liver as the central organ in lipid metabolism and synthesis of coagulation factors. To identify drug-specific molecular responses and overlap between the various treatments, the total number of differentially expressed genes (DEG) was assessed and a Venn-diagram was constructed comprising all DEG compared to control group. Both ponatinib and nilotinib displayed a dose-dependent increase in the total number of DEG as compared to control and the molecular response of the high-dose ponatinib had more overlapping genes with imatinib than nilotinib (Figure S2; Data Sheet S1**)**.

General categorization of biological functions showed that all anti-CML drugs affect canonical pathways associated with mitochondrial dysfunction and oxidative phosphorylation, most likely induced by oxidative stress and leading to reduced energy production, and processes involved in protein synthesis and cell growth (EIF2 signaling), confirming the target-related molecular responses of anti-CML drugs. ( Figure S3; Data Sheet S1).

**Figure 7:**
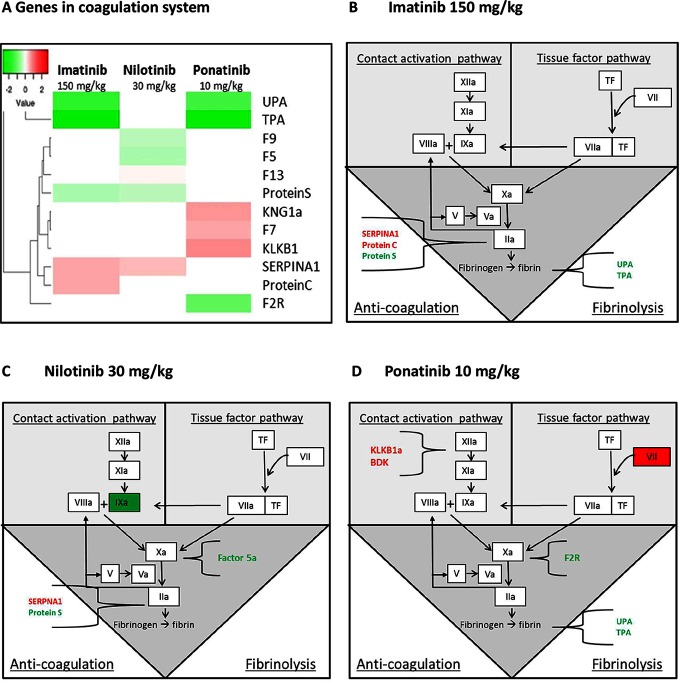
Genes in the coagulation system regulated by imatinib, nilotinib and ponatinib. The heat map shows all significantly (<0.01) upregulated (red) and downregulated (green) genes involved in coagulation of mice treated with imatinib (150 mg/kg), nilotinib (30 mg/kg) or ponatinib (10 mg/kg) as compared to control mice **(A)**. Imatinib regulates genes involved in anti-coagulation and fibrinolysis **(B)**. Factor 9a and 5a were down-regulated and SERPINA1 up-regulated by nilotinib **(C)**. Ponatinib showed the most adverse profile with upregulation of genes in both the contact activation and tissue factor pathway, together with downregulation of genes involved in fibrinolysis **(D).***p*-values of <0.01 were used as cut-off (*n* = 8 per group).

The processes relevant to (cardio)vascular (side) effects of the anti-CML drugs are highlighted in Figure S4; Data Sheet S1 and Figure 7**.** Although gene expression data from the liver cannot be directly extrapolated to atherosclerosis signaling in the vascular wall, gene expression profiles of different organs have affiliation with each other and may be predictive for these biological processes. Therefore, the transcriptome data of the liver as predicting organ are given. As compared to the other TKI’s, imatinib showed the most pronounced effects on atherosclerosis signaling, with favorable regulation of genes involved in cell adhesion (*Integrin β2 and α4, Icam1, Vcam1, Psgl-1*), macrophage activation (C*d40, Tnfrsf14, Scara1, Nfκb*), lipid regulation (L*pl, Apoa1, Apoa2, Apoc2, Apoc4, Pla2g7* ), inflammatory processes (*Cd40, Nfκb, Il1a, Tnfrsf14, Icam1, Vcam1)* and genes related to extracellular matrix modulation (*Col1a2, Col3a1, Col1a1, Mmp13, Tgf-β*) (Figure S4 A–B data; Data Sheet S1). Ponatinib showed similar effects, but to a lesser extent, whereas these effects were not observed after nilotinib treatment (Figure S4 A; Data Sheet S1).

As the site of synthesis of a large number of coagulation factors, the liver plays an important role in the regulation of hemostatic and thrombotic processes. Although all three TKIs to some extent affected the coagulation pathways, ponatinib had the most adverse profile (Figure 7**)**. Ponatinib increased the gene expression of members of the intrinsic or contact activation pathway, *Kng1a* and *Klkb1*, mainly involved in growth of a thrombus, and of the extrinsic or tissue factor pathway *F7*, involved in initiation of thrombus formation, and decreased gene expression of *Upa* and *Tpa*, both involved in fibrinolysis. Nilotinib showed down-regulation of the expression of *F5*, *F9*, and *Protein S*, while *Serpina1 (PAI-1)* was up-regulated. Imatinib down-regulated the expression of *Upa, Tpa* and *Protein S* and up-regulated *Serpina1* and *Protein C*. This analysis demonstrates that among the three anti-CML drugs investigated ponatinib most prominently induces adverse alterations in the gene expression of coagulation factors in both the intrinsic and extrinsic pathway, which may lead to a state of hypercoagulability.

### Coagulation

####  Ponatinib Increases Plasma Factor VII and Nilotinib Increases Factor VIIa Activity

**Figure 8:**
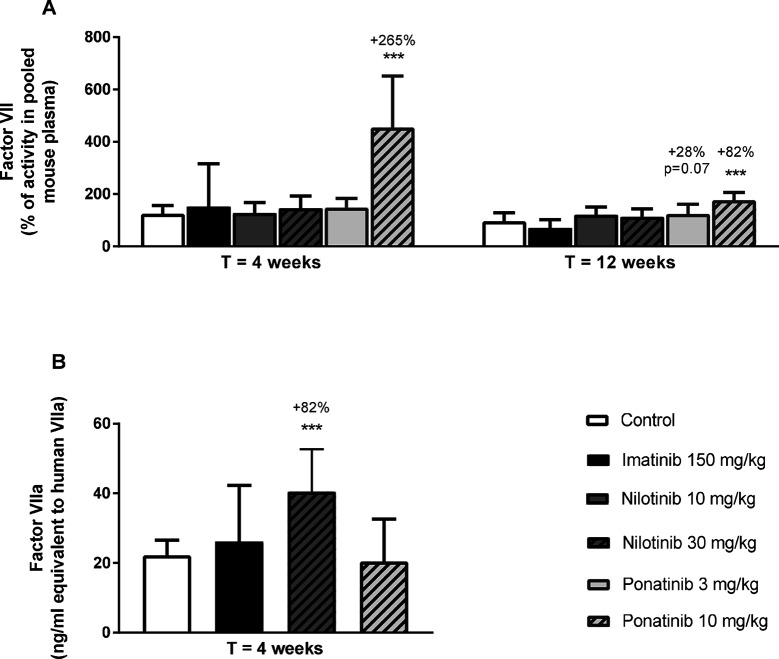
Ponatinib increases factor VII and nilotinib increases factor VIIa activity. Factor VII was measured after 4 and 12 weeks of treatment as the percentage of activity in reference pooled mouse plasma **(A).** Factor VIIa activity was measured after 4 weeks of treatment **(B)**. ***p < 0.001. Data are presented as means ± SD (*n* = 6–20 per group).

Next, we measured total factor VII coagulant activity (FVII) and VIIa activity (FVIIa) in plasma. Ponatinib increased FVII after 4 weeks (10 mg/kg, +265%, *p* < 0.001) and 12 weeks of treatment (3 mg/kg, +28%, *p* = 0.07; 10 mg/kg + 82%, *p* < 0.001) (Figure 8A). In addition, nilotinib increased the activity of FVIIa at 4 weeks by 82% (30 mg/kg, *p* < 0.001) (Figure 8B). Together, these data confirm our findings on gene expression analysis and reveal the pro-thrombotic characteristics of nilotinib and ponatinib.

## Discussion

This is the first study that compared the effect of a first, second and third generation BCR-ABL1 tyrosine kinase inhibitor on (cardio)vascular risk factors and atherosclerosis. Imatinib and ponatinib decreased plasma cholesterol and atherosclerosis, while nilotinib and ponatinib activated coagulation. The pharmacokinetic data we provide enabled us to use drug exposures translatable to CML-patients and can be used to optimize future TKI research. In addition, we provide a robust data set obtained by gene expression and pathway analysis of the liver, which predicted that ponatinib may lead to a pro-coagulant state by adversely affecting coagulation factors of both the contact activation (intrinsic) and tissue factor (extrinsic) pathways, which was confirmed by increased levels of the coagulation factor VII. In addition, nilotinib increased activity of FVIIa. These findings can be used by clinicians to carefully monitor coagulation parameters in CML-patients to predict risk of cardiovascular events.

The choice to perform this study in a non-leukemic mouse model has several reasons. First, there is, inherent to the diagnosis and progression of the disease, a shortage of suitable high quality plasma samples of CML patients collected at both baseline and follow-up under similar conditions. Second, CML affects both metabolic (10) and coagulation (11) parameters which makes it difficult to elucidate the role of TKI treatment on the reported VAEs independently of the underlying disease. Last, we were able to investigate a broad range of parameters, including atherosclerosis and gene expression and pathway analysis of the liver, which is not possible in CML-patients.

Imatinib and ponatinib, but not nilotinib, decreased plasma cholesterol contained in the pro-atherogenic apoB-containing lipoproteins. Cholesterol reduction and even normalization in hypercholesterolemic CML-patients is repeatedly described in retrospective studies with CML-patients for imatinib (30–32, 37) and is consistent with our findings. Data on ponatinib are scarce (37) and it is unclear whether nilotinib affects plasma cholesterol in CML-patients. Some studies reported increased plasma cholesterol (5, 8, 33, 37), whereas others question this (38). These opposing findings may be explained by the response to the underlying disease. It should be noted that nilotinib is often prescribed as a second-line treatment after resistance to imatinib. Reduced caloric intake induced by the leukemia and increased energy requirements imposed by tumor growth may result in lower cholesterol levels at baseline, while a positive response to treatment is often accompanied by increased cholesterol levels in oncologic patients (39). This response-related cholesterol elevation may be abolished by the cholesterol-lowering effects of imatinib and ponatinib *per se* as found in our study, resulting in decreased (imatinib) or normalized (ponatinib) plasma cholesterol levels in CML patients.

Several mechanisms are involved in cholesterol homeostasis, including intestinal uptake, hepatic uptake and secretion as lipoproteins, synthesis and storage, and fecal excretion. The decreased hepatic lipid content in imatinib and ponatinib treated mice (Figure 2) points to a shortage of cholesterol in the liver and suggests that not lipoprotein clearance, but VLDL production and/or intestinal absorption of cholesterol are affected by imatinib and ponatinib. Indeed, when myeloid tumor cells are treated with imatinib, *de novo* fatty acid synthesis is reduced, pointing towards decreased VLDL particle production (40). However, besides the shared activity of TKIs used for CML-treatment against the BCR-ABL1 tyrosine kinase, the potency and activity to affect off-target kinases differs markedly, and thus different processes may be involved. To our knowledge, no *in vivo* studies are available that investigated the effects of TKI treatment on cholesterol and lipoprotein metabolism, and functional studies are required.

Important observations from our study are the reduced development of atherosclerosis by imatinib and ponatinib which was correlated to decreased plasma cholesterol levels, and the increased plaque stability induced by imatinib, which has not been reported previously by others. There are no reports that describe the effect of ponatinib on atherosclerosis development in an animal model, and there are inconsistent reports on imatinib and nilotinib. In line with our findings, imatinib reduced atherosclerosis in STZ-induced diabetic ApoE^−/−^ mice (41) and high fat fed ApoE^−/−^ mice (42), though lesion reduction was independent of plasma cholesterol lowering and attributed to vascular wall remodeling and reduced inflammation. Interestingly, and in contrast with previous (41–43) and the present findings, Hadzijusufovic et al (43). did not find an effect of imatinib on atherosclerosis in ApoE^−/−^ mice, but reported increased atherosclerosis by nilotinib. In addition, a direct pro-atherogenic effect on human endothelial cells was found, as shown by upregulation of adhesion factors ICAM-1, E-selectin and VCAM-1 (44), which is in line with the increase of markers of endothelial activation found in CML patients treated with nilotinib (45). Unfortunately, no data on plasma cholesterol and markers of endothelial activation in the mice were provided. We do not have a clear explanation for the discrepancy with our findings, but the use of different animal models and dosages, as well as the underlying disease may play a role.

Ponatinib increased plasma E-selectin and urinary albumin:creatinin in some animals, suggesting endothelial activation and potential endothelial dysfunction, wherein aberrant angiogenesis might be involved. Indeed, an *in vitro* study using HUVECs demonstrated the potential of ponatinib to reduce endothelium viability, and to induce apoptosis, reduce migration, inhibit tube formation, and to negatively affect endothelial progenitor cell function, all important for angiogenesis (46). In addition, ponatinib reduced vWF expression on lung endothelial cells in rats (47), which is an interesting finding, because vWF is not only a specific marker for ECs, but also functions in coagulation.

Although there were no clinical signs of thrombosis or bleeding in our long term study, an unbiased and exploratory transcriptome analysis revealed that ponatinib treatment lead to a pro-thrombotic state by affecting important players in the activation of the coagulation pathway. Ponatinib increased gene expression of *Klkb1*, *Kng1a* (part of the intrinsic or contact activation pathway) and *F7* (part of the extrinsic or tissue factor pathway) and decreased expression of *Upa* and *Tpa*, which function in resolution of thrombi by fibrinolysis. Consistent with the increased gene expression, plasma levels of factor VII were increased by 265%. Nilotinib had less pronounced effects on gene expression of coagulation factors but increased activity of FVIIa by 82%. Using a different experimental design, Alhawiti et al (45). recently reported that a single dose of nilotinib but not imatinib increased platelet aggregation and thrombus growth *ex vivo* and *in vivo* in mice, and increased *ex vivo* platelet adhesion and thrombin generation in CML-patients receiving nilotinib (45). On the other hand, Loren et al (48). demonstrated that ponatinib, but not imatinib and nilotinib, inhibited *ex vivo* human platelet activation, spreading and aggregation, and hypothesized that the cardiovascular events observed in patients treated with ponatinib may be the result of effects on other organs and cell types. Indeed, we show that FVII is involved, which is produced by the liver, and is an important factor in the coagulation pathway. Mice lacking FVII have delayed thrombus formation (49) and pharmacological doses of rFVIIa induce hemostasis in severe hemophilia and in non-hemophilia patients with profuse, heavy bleeding (50). Collectively, these data indicate that nilotinib and ponatinib can both potentiate a pro-thrombotic state via different mechanisms of action.

The presence of one or more risk factors for (cardio)-metabolic disease together with the increased platelet aggregation and increased plasma activity of factor VII/VIIa may contribute to the onset of thrombosis, especially when combined with increased levels of tissue factor (TF), which activates the tissue factor pathway (Figure 7). Hypercoagulability has been described in a variety of malignancies, including hematological malignancies (51, 52), and many tumor cells express high levels of TF, the primary initiator of the extrinsic coagulation pathway (53). Therefore, we propose that nilotinib and ponatinib induce (athero) thrombosis in a subgroup of CML-patients through a combination of (cardio)-metabolic risk factors, enhanced levels of TF and increased plasma levels of coagulation factors. Our findings can be used to develop a multivariate risk model for CVD in CML patients, which include (cardio) vascular risk factors and coagulation parameters at baseline and during treatment, facilitating an early detection strategy for patients prone to cardiovascular events, which will improve therapy decision and patient care.

In conclusion, using a comprehensive approach to measure the cardiovascular effects of various BCR-ABL inhibitors, we demonstrate that first, second and third generation BCR-ABL inhibitors have very distinct effects on lipid metabolism, blood coagulability and atherosclerosis. The first-generation inhibitor imatinib was proven safe, with evident benefit for plasma lipid concentrations, atherosclerotic plaque size and stability. The third-generation inhibitor ponatinib showed similar, albeit less pronounced effects on lipid concentrations and atherosclerosis, but also showed a hypercoagulable phenotype. These data perfectly match retrospective clinical observations on cardiovascular effects of BCR-ABL inhibitors, and besides providing a biological basis for these observations, may well contribute to safer application of these drugs in the future.

## Ethics Statement

Animal experiments were approved by the Institutional Animal Care and Use Committee of The Netherlands Organization for Applied Research under registration number 3557.

## Author Contributions

MP performed the research, analyzed the data and wrote the paper. EP designed the research, performed the research and analyzed the data. LV and MC performed and analyzed the gene expression analysis. CK developed the plasma coagulation factor assays and analyzed the plasma samples. RG designed the research and analyzed the PK data. JA analyzed the data and edited the paper. WJ edited the paper. HP designed the research, analyzed the data and edited the paper.

## Conflict of Interest Statement

CK has ownership interests in ‘Good Biomarker Sciences’. RG is an employee at Bristol-Meyers Squibb, New York, USA. WJ received research grants from and has spoken at (CME-accredited) meetings sponsored by Amgen, Astellas, Astra-Zeneca, Daiichi Sankyo, Lilly, Merck-Schering-Plough, Pfizer, Roche, Sanofi-Aventis, the Netherlands Heart Foundation, the Interuniversity Cardiology Institute of the Netherlands, and the European Community Framework KP7 Program.

The remaining authors declare that the research was conducted in the absence of any commercial or financial relationships that could be constructed as a potential conflict of interest.

## Abbreviations

ALT: alanine transaminase
AST: aspartate transaminase
BAL: broncho-alveolar lavage
BID: twice a day
CML: chronic myeloid leukemia
DEGs: differentially expressed genes
HPTLC: High-performance thin-layer chromatography
MCP-1: monocyte chemoattractant protein-1
PAOD: progressive arterial occlusive disease
PBMCs: peripheral blood mononuclear cells
PK: pharmacokinetic
QD: once daily
SAA: serum amyloid A
TC: total cholesterol
TG: triglycerides
TKIs: tyrosine kinase inhibitors
VAEs: vascular adverse effects
