# Supplementary material for: The BCR-ABL1 Inhibitors Imatinib and Ponatinib Decrease Plasma Cholesterol and Atherosclerosis, and Nilotinib and Ponatinib Activate Coagulation in a Translational Mouse Model
Source: Front Cardiovasc Med. 2018 Jun 12;5:55. doi: 10.3389/fcvm.2018.00055 (PMC6005845; doi:10.3389/fcvm.2018.00055)

Supplementary Material

1 Supplementary introduction and methods

1.2 Introduction

Background of the APOE*3Leiden.CETP mouse and response to treatment

The APOE*3Leiden.CETP transgenic mouse model is an established model for hyperlipidemia and the development of atherosclerosis and was developed by cross-breeding of the APOE*3Leiden transgenic mice and human cholesterol ester transfer protein (CETP) transgenic mice.

The APOE*3Leiden mouse was initially developed as an animal model for Familial Dysbetalipoproteinemia (FD) or type III hyperlipoproteinemia, which is characterized by elevated levels of cholesterol and an increased ratio of cholesterol to triglycerides in the very-low-density-lipoprotein (VLDL) and intermediate-density lipoprotein (IDL) fractions, resulting in the appearance of β-VLDL particles[1 2]. Similarly as in FD patients, in E3L and E3L.CETP mice as a model for mixed dyslipoproteinemia, a major part of plasma cholesterol is contained in the VLDL and VLDL-remnant particles, leading to formation of β-VLDL particles, which further increases after cholesterol feeding.

E3L mice were generated by the introduction of a DNA-construct containing the human *apoe*3Leiden* and *apoc1* genes[1]. The primary effect of the dominant E*3-Leiden mutation is an impaired clearance of triglyceride-rich lipoproteins (chylomicron- and VLDL-remnants) caused by a reduced affinity of the apolipoprotein E*3Leiden for the LDLR[3], whereas overexpression of apolipoprotein C1 inhibits lipolysis. While normal wild-type mice have a

very rapid clearance of apoB-containing lipoproteins, APOE*3Leiden mice show an impaired clearance and are thereby mimicking the slow clearance observed in humans, particularly in patients with FD or type III hyperlipoproteinemia. Mice in contrast to humans do not possess CETP. CETP transgenic mice express the human *cetp* gene under control of its natural flanking regions[4]. CETP transfers cholesteryl ester from HDL to the apoB-containing lipoproteins in exchange for triglycerides, resulting in a more human-like lipoprotein metabolism in APOE*3Leiden.CETP mice.

APOE*3Leiden.CETP mice are prone to develop hyperlipidemia and atherosclerosis upon feeding a western type diet containing saturated fat and cholesterol[5 6]. The model responds to all hypolipidemic drugs currently used in the clinic, such as statins, fibrates, ezetimibe, niacin and PCSK9 mAbs, at similar dosages and in a similar way to humans[7-16]. Furthermore, the model responds well to blood pressure lowering drugs[17 18].

In the present study, female APOE*3Leiden.CETP mice are used because they are more susceptible to cholesterol-containing diets by having higher plasma cholesterol and triglyceride levels[19] and therefore develop atherosclerotic lesions.

1.2 Materials and methods

Animals

Female APOE*3Leiden.CETP transgenic mice (9 to 14 weeks of age) were used. During the study, mice were housed under standard conditions with a 12-h light-dark cycle and had free access to food and water. Body weight, food intake and clinical signs of behavior were monitored regularly during the study. For the (cardio)vascular risk factor-atherosclerosis study, the number of animals per group was calculated using a probability of 0.05. Based on our experience from previous studies, we expected to have a variance of 15% (sigma 40%) in

plasma lipids and a minimal effect of treatment of 30%, resulting in 15 animals per group. Animal experiments were approved by the Institutional Animal Care and Use Committee of The Netherlands Organization for Applied Research under registration number 3557.

PK analysis and plasma drug concentrations

EDTA plasma samples collected during the 24-hour PK study and at week 16 of the (cardio)vascular risk factor-atherosclerosis study were subsequently stored at -80°C until analysis. Thawed plasma samples (50μL) were de-proteinized with two volumes of acetonitrile containing an appropriate src-inhibitor chemotype as internal standard for each analyte. Following deproteinization, a 5μL portion of clear supernatant was then subjected to solvent gradient separation in an Agilent 1100 series HPLC system interfaced to a Micromass triple-quadrupole mass spectrometer, which was operated in the positive ion electrospray MRM mode to obtain daughter ions for quantitation: Ion transitions used for quantitation were as follows: imatinib [494.3 -> 394.2], nilotinib [530.2 -> 289.2], ponatinib [533.3 -> 260.3]. Standard curves ranging from 1 nM to 6 uM were fitted with a quadratic regression weighted by reciprocal concentration (1/x). LLOQ for the purposes of this assay was between 1 and 2 nM for all compounds analyzed. QC samples at three levels in the range of the standard curve were used to accept individual analytical sets, and all results were calculated as the mean of triplicate determinations ± standard error. T_{max}, C_{max} and AUCs were calculated using the software Berkeley Madonna (version 8.3.18).

Biochemical analyses and blood pressure

EDTA plasma samples were collected throughout the study. Plasma cholesterol and triglycerides were determined every 4 weeks using enzymatic kits (Roche/Hitachi) according

to the manufacturer's protocols and average plasma levels over week 4 to 16 were calculated. HDL-C was measured after precipitation as described previously[20]. The distribution of cholesterol over plasma lipoproteins was determined in group wise-pooled plasma by fast protein liquid chromatography (FPLC)[9]. The inflammatory markers SAA, E-selectin and MCP-1 were measured using the ELISA kits from Tridelta (SAA) and R&D (MCP-1, E-selectin) according to the manufacturer's instruction. Plasma ALT and AST were determined using a spectrophotometric assay (Boehringer Reflotron system) in group wise-pooled samples. Blood pressure (SBP, DBP) and heart rate were measured using the tail cuff method in 8 mice per group at 2 and 15 weeks[18].

Hepatic lipid analysis

Livers were isolated and partly homogenized (30 s at 5000 rpm) in saline (~10% wet wt/vol) using a mini-bead beater (Biospec Products, Inc., Bartlesville, OK). Lipids were extracted as described[21] previously and separated by high-performance thin-layer chromatography. Lipid spots were stained with color reagent (5 g MnCl2.4H2O, 32 ml 95–97% H2SO4 added to 960 ml of CH3OH:H2O 1:1 vol/vol) and quantified using TINA version 2.09 software (Raytest, Straubenhardt, Germany).[21]

BAL and urinary albumin:creatinin

The lungs were flushed two times with 750 µl PBS into the trachea using a BD 20G angio-catheter to collect broncho-alveolar lavage (BAL) fluid. Protein and albumin content in BAL fluid were determined in the supernatant using the protein determination kit from Pierce and the mouse albumin ELISA kit (ALPCO, Salem, USA). Urinary albumin and creatinin levels were determined using the mouse albumin ELISA kit (ALPCO, Salem, USA) and the creatinin kit (Exocell, Philadelphia, USA) according to manufacturer's instruction.

Flow cytometric analysis

White cell profiling was performed via FACS using the BD FACS Canto II apparatus (Becton Dickinson, Franklin Lakes, New Jersey, USA). After 12 weeks of treatment, peripheral blood mononuclear cells (PBMCs) were isolated from fresh blood samples of 8 mice per group, and were sorted into CD11b+/CD11c- (monocytes), and further divided into CD11b+/Ly6C^{low} and CD11b+/Ly6C^{high} monocytes. The following conjugated monoclonal antibodies, all from eBiosciences, were used: CD11b-FITC, CD11c-PE/Cy7, Ly6C-APC.

Coagulation factor VII and VIIa

Total clotting FVII and FVIIa activity were measured on a STA compact apparatus (Diagnostica Stago Inc. Parsippany, NJ). For the determination of total clotting FVII an one stage Prothrombin assay with Dade Innovin PT reagent (Siemens) and Hemoclot FVII reagent (Hyphen Biomed) as deficient agent were used and calibration was performed with pooled normal mouse plasma. Staclot VIIa rTF (Diagnostica Stago Inc.) and Hemoclot FVII reagent (Hyphen Biomed) were used to determine FVIIa activity, calibrated with Novoseven® (Novonordisk).

Histological assessment of lung morphology and atherosclerosis

Tissues were isolated, fixed in formalin, and embedded in paraffin. The caudal lung was cross sectioned (3 µm thick) and stained with hematoxylin-eosin (HE), Sirius Red for collagen, and with isolectin B4 (1:50; Sigma-Aldrich, Missouri, USA) for endothelial cells. Hearts were sectioned perpendicular to the axis of the aorta, starting within the heart and working in the direction of the aortic arch. Once the aortic root was identified by the appearance of aortic

valve leaflets and smooth muscle cells instead of collagen-rich tissue, serial cross sections (5 µm thick with intervals of 50 µm) were taken and mounted on AAS-coated slides. These sections were stained with hematoxylin-phloxine-saffron (HPS) for histological analysis. For each mouse, atherosclerosis was measured in 4 subsequent cross sections. Each section consisted of 3 segments. The average total lesion area per cross section was then calculated[20 22]. For determination of lesion severity the lesions were classified into five categories according to the American Heart Association classification[23]: 0) no lesion I) early fatty streak, II) regular fatty streak, III) mild plaque, IV) moderate plaque, and V) severe plaque. The percentage of each lesion type was calculated, where type I-III lesions were classified as mild lesions and type IV-V lesions were classified as severe lesions[20 22]. In the aortic root, lesion composition was determined for the severe lesions (type IV-V) as a percentage of lesion area after immunostaining with anti-human alpha-actin (1:400; PROGEN Biotechnik GmbH, Germany) for smooth muscle cells (SMC), and anti-mouse Mac-3 (1:50; BD Pharmingen, the Netherlands) for macrophages followed by Sirius Red staining. After Sirius Red staining for collagen, color intensity was determined in ImageJ and the used threshold was confirmed by evaluation of the sections under polarized light[24]. Necrotic area and cholesterol clefts were measured after HPS staining[13 20 22]. Lesion stability index was calculated as described previously[13 20]. In each segment used for lesion quantification, the number of monocytes adhering to the endothelium were counted after immunostaining with AIA 31240 antibody (1:1000; Accurate Chemical and Scientific, New York, New York, USA)[14 20].

Gene expression analysis

Messenger RNA was isolated from liver of 8 mice per group, using the NEBNext Ultra RNA sample Prep Kit. After fragmentation of the mRNA, cDNA synthesis was performed. The

quality and yield after sample preparation was measured with the Fragment Analyzer. Clustering and DNA sequencing was performed using the Illumina Nextseq 500. The genome reference and annotation file Mus_Musculus.GRCm38 was used for analysis in FastA and GTF format. The reads were aligned to the reference sequence using Tophat 2.0.14 combined with Bowtie 2.1.0, and based on the mapped read locations and the gene annotation HTSeq-count version 0.6.1p1 was used to count how often a read was mapped on the transcript region. Calculated P-values <0.01 were used as threshold for significance. Selected differentially expressed genes (DEGs) were used as an input for pathway analysis through Ingenuity Pathway Analysis (IPA) suite (www.ingenuity.com, accessed 2015). Gene set enrichment analysis was used to highlight the most important processes and pathways. Relevance of these pathways and processes is indicated as p-value and visualized in a graph by calculating the –log(p-value).

1.3 References

1. van den Maagdenberg AM, Hofker MH, Krimpenfort PJ, de Bruijn I, van Vlijmen B, van der Boom H, Havekes LM, Frants RR. Transgenic mice carrying the apolipoprotein E3-Leiden gene exhibit hyperlipoproteinemia. *J Biol Chem* 1993;268(14):10540-5.
2. de Knijff P, van den Maagdenberg AM, Stalenhoef AF, Leuven JA, Demacker PN, Kuyt LP, Frants RR, Havekes LM. Familial dysbetalipoproteinemia associated with apolipoprotein E3-Leiden in an extended multigeneration pedigree. *J Clin Invest* 1991;88(2):643-55.
3. Wardell MR, Weisgraber KH, Havekes LM, Rall SC, Jr. Apolipoprotein E3-Leiden contains a seven-amino acid insertion that is a tandem repeat of residues 121-127. *J Biol Chem* 1989;264(35):21205-10.
4. Jiang XC, Agellon LB, Walsh A, Breslow JL, Tall A. Dietary cholesterol increases transcription of the human cholesteryl ester transfer protein gene in transgenic mice. Dependence on natural flanking sequences. *J Clin Invest* 1992;90(4):1290-5.
5. van Vlijmen BJ, van den Maagdenberg AM, Gijbels MJ, van der Boom H, HogenEsch H, Frants RR, Hofker MH, Havekes LM. Diet-induced hyperlipoproteinemia and atherosclerosis in apolipoprotein E3-Leiden transgenic mice. *J Clin Invest* 1994;93(4):1403-10.
6. Westerterp M, van der Hoogt CC, de Haan W, Offerman EH, Dallinga-Thie GM, Jukema JW, Havekes LM, Rensen PC. Cholesteryl ester transfer protein decreases high-density lipoprotein and severely aggravates atherosclerosis in APOE*3-Leiden mice. *Arterioscler Thromb Vasc Biol* 2006;26(11):2552-9.
7. Kleemann R, Verschuren L, van Erk MJ *et al.* Atherosclerosis and liver inflammation induced by increased dietary cholesterol intake: a combined transcriptomics and metabolomics analysis. *Genome Biol* 2007;8(9):R200.
8. de Haan W, van der Hoogt CC, Westerterp M, Hoekstra M, Dallinga-Thie GM, Princen HM, Romijn JA, Jukema JW, Havekes LM, Rensen PC. Atorvastatin increases HDL cholesterol by reducing CETP expression in cholesterol-fed APOE*3-Leiden.CETP mice. *Atherosclerosis* 2008;197(1):57-63.
9. Kooistra T, Verschuren L, de Vries-van der Weij J, Koenig W, Toet K, Princen HM, Kleemann R. Fenofibrate reduces atherogenesis in ApoE*3Leiden mice: evidence for multiple antiatherogenic effects besides lowering plasma cholesterol. *Arterioscler Thromb Vasc Biol* 2006;26(10):2322-30.
10. van der Hoogt CC, de Haan W, Westerterp M, Hoekstra M, Dallinga-Thie GM, Romijn JA, Princen HM, Jukema JW, Havekes LM, Rensen PC. Fenofibrate increases HDL-cholesterol by reducing cholesteryl ester transfer protein expression. *J Lipid Res* 2007;48(8):1763-71.
11. Verschuren L, Radonjic M, Wielinga PY, Kelder T, Kooistra T, van Ommen B, Kleemann R. Systems biology analysis unravels the complementary action of combined rosuvastatin and ezetimibe therapy. *Pharmacogenet Genomics* 2012;22(12):837-45.
12. Gierman LM, Kuhnast S, Koudijs A, Pieterman EJ, Kloppenburg M, van Osch GJ, Stojanovic-Susulic V, Huizinga TW, Princen HM, Zuurmond AM. Osteoarthritis development is induced by increased dietary cholesterol and can be inhibited by atorvastatin in APOE*3Leiden.CETP mice--a translational model for atherosclerosis. *Ann Rheum Dis* 2014;73(5):921-7.
13. Kuhnast S, Louwe MC, Heemskerk MM, Pieterman EJ, van Klinken JB, van den Berg SA, Smit JW, Havekes LM, Rensen PC, van der Hoorn JW, Princen HM, Jukema JW. Niacin Reduces Atherosclerosis Development in APOE*3Leiden.CETP Mice Mainly by Reducing NonHDL-Cholesterol. *PLoS One* 2013;8(6):e66467.
14. Kuhnast S, van der Hoorn JW, Pieterman EJ, van den Hoek AM, Sasiela WJ, Gusarova V, Peyman A, Schafer HL, Schwahn U, Jukema JW, Princen HM. Alirocumab inhibits atherosclerosis, improves the plaque morphology, and enhances the effects of a statin. *J Lipid Res* 2014;55(10):2103-12.

15. van den Hoek AM, van der Hoorn JW, Maas AC, van den Hoogen RM, van Nieuwkoop A, Droog S, Offerman EH, Pieterman EJ, Havekes LM, Princen HM. APOE*3Leiden.CETP transgenic mice as model for pharmaceutical treatment of the metabolic syndrome. *Diabetes Obes Metab* 2014;16(6):537-44.
16. Ason B, van der Hoorn JW, Chan J *et al.* PCSK9 inhibition fails to alter hepatic LDLR, circulating cholesterol, and atherosclerosis in the absence of ApoE. *J Lipid Res* 2014;55(11):2370-9.
17. van der Hoorn JW, Kleemann R, Havekes LM, Kooistra T, Princen HM, Jukema JW. Olmesartan and pravastatin additively reduce development of atherosclerosis in APOE*3Leiden transgenic mice. *J Hypertens* 2007;25(12):2454-62.
18. Kuhnast S, van der Hoorn JW, van den Hoek AM, Havekes LM, Liau G, Jukema JW, Princen HM. Aliskiren inhibits atherosclerosis development and improves plaque stability in APOE*3Leiden.CETP transgenic mice with or without treatment with atorvastatin. *J Hypertens* 2012;30(1):107-16.
19. van Vlijmen BJ, van 't Hof HB, Mol MJ, van der Boom H, van der Zee A, Frants RR, Hofker MH, Havekes LM. Modulation of very low density lipoprotein production and clearance contributes to age- and gender- dependent hyperlipoproteinemia in apolipoprotein E3-Leiden transgenic mice. *J Clin Invest* 1996;97(5):1184-92.
20. Kuhnast S, van der Tuin SJ, van der Hoorn JW, van Klinken JB, Simic B, Pieterman E, Havekes LM, Landmesser U, Luscher TF, Willems van Dijk K, Rensen PC, Jukema JW, Princen HM. Anacetrapib reduces progression of atherosclerosis, mainly by reducing non-HDL-cholesterol, improves lesion stability and adds to the beneficial effects of atorvastatin. *Eur Heart J* 2015;36(1):39-48.
21. Post SM, Zoeteweij JP, Bos MH, de Wit EC, Havinga R, Kuipers F, Princen HM. Acyl-coenzyme A:cholesterol acyltransferase inhibitor, avasimibe, stimulates bile acid synthesis and cholesterol 7alpha-hydroxylase in cultured rat hepatocytes and in vivo in the rat. *Hepatology* 1999;30(2):491-500.
22. Delsing DJ, Offerman EH, van Duyvenvoorde W, van Der Boom H, de Wit EC, Gijbels MJ, van Der Laarse A, Jukema JW, Havekes LM, Princen HM. Acyl-CoA:cholesterol acyltransferase inhibitor avasimibe reduces atherosclerosis in addition to its cholesterol-lowering effect in ApoE*3-Leiden mice. *Circulation* 2001;103(13):1778-86.
23. Stary HC, Chandler AB, Dinsmore RE, Fuster V, Glagov S, Insull W, Jr., Rosenfeld ME, Schwartz CJ, Wagner WD, Wissler RW. A definition of advanced types of atherosclerotic lesions and a histological classification of atherosclerosis. A report from the Committee on Vascular Lesions of the Council on Arteriosclerosis, American Heart Association. *Arterioscler Thromb Vasc Biol* 1995;15(9):1512-31.
24. Landlinger C PM, Juno C, et al. The AT04A vaccine against proprotein convertase subtilisin/kexin type 9 reduces total cholesterol, vascular inflammation, and atherosclerosis in APOE*3Leiden.CETP mice. . *Eur Heart J* 2017.

2 Supplementary Figures and Tables

2.1 Supplementary Tables

Table S1. Safety aspects of TKI treatment. Body weight , food intake (per cage) plasma ALT (pooled), and plasma AST (pooled) at baseline and after 16 weeks of treatment with imatinib (150 mg/kg, BID), nilotinib (10 and 30 mg/kg) or ponatinib (3 and 10 mg/kg).

	**Dose** *mg/kg*	**Body weight** *gram*	**Food intake** *gram/mouse/day*	**ALT** *U/L*	**AST** *U/L*
**Baseline**	-	21.6 ± 1.7	3.0	57	163
**Control**	-	23.3 ± 2.4	2.5	54	224
**Imatinib**	150	22.1 ± 0.8	2.6	31	147
**Nilotinib**	10	22.5 ± 1.5	2.3	64	210
	30	22.2 ± 1.3	2.4	81	221
**Ponatinib**	3	22.5 ± 2.3	2.4	44	196
	10	22.3 ± 2.8	2.3	31	207

ALT, Alanine transaminase; AST, aspartate transaminase (n = 2-4 mice per cage) (n = 13-15 per group). Data are presented as means ± SD (n=13-15 per group) or means (cage or group level).

Table S2. Imatinib and ponatinib reduce the number of live PBMCs and all TKIs decrease the fraction of pro-inflammatory CD11b+Ly6C^{high} monocytes. After 12 weeks of treatment with imatinib (150 mg/kg, BID), nilotinib (10 and 30 mg/kg) or ponatinib (3 and 10 mg/kg) FACS analysis was performed.

	Dose	Number of live/single PBMCs per mL blood	% of monocyte population	
	mg/kg	*(*10^6/mL)*	• CD11b+Ly6C^{high}	• CD11b+Ly6C^{low}
**Control**	-	6.8 ± 1.3	19.0 ± 4.4	71.7 ± 3.9
**Imatinib**	150	3.9 ± 1.3**	11.0 ± 3.6**	69.1 ± 5.9
**Nilotinib**	10	6.5 ± 2.4	27.5 ± 4.3	61.4 ± 4.8
	30	6.4 ± 1.4	11.4 ± 3.8*	73.2 ± 4.9
**Ponatinib**	3	7.1 ± 1.7	26.1 ± 2.9	58.4 ± 2.5
	10	3.8 ± 0.8**	12.6 ± 3.1*	69.1 ± 6.4

PBMC, peripheral blood mononuclear cell. *P<0.05, **P<0.01 as compared to control. Data are presented as means ± SD (n=7-8 per group).

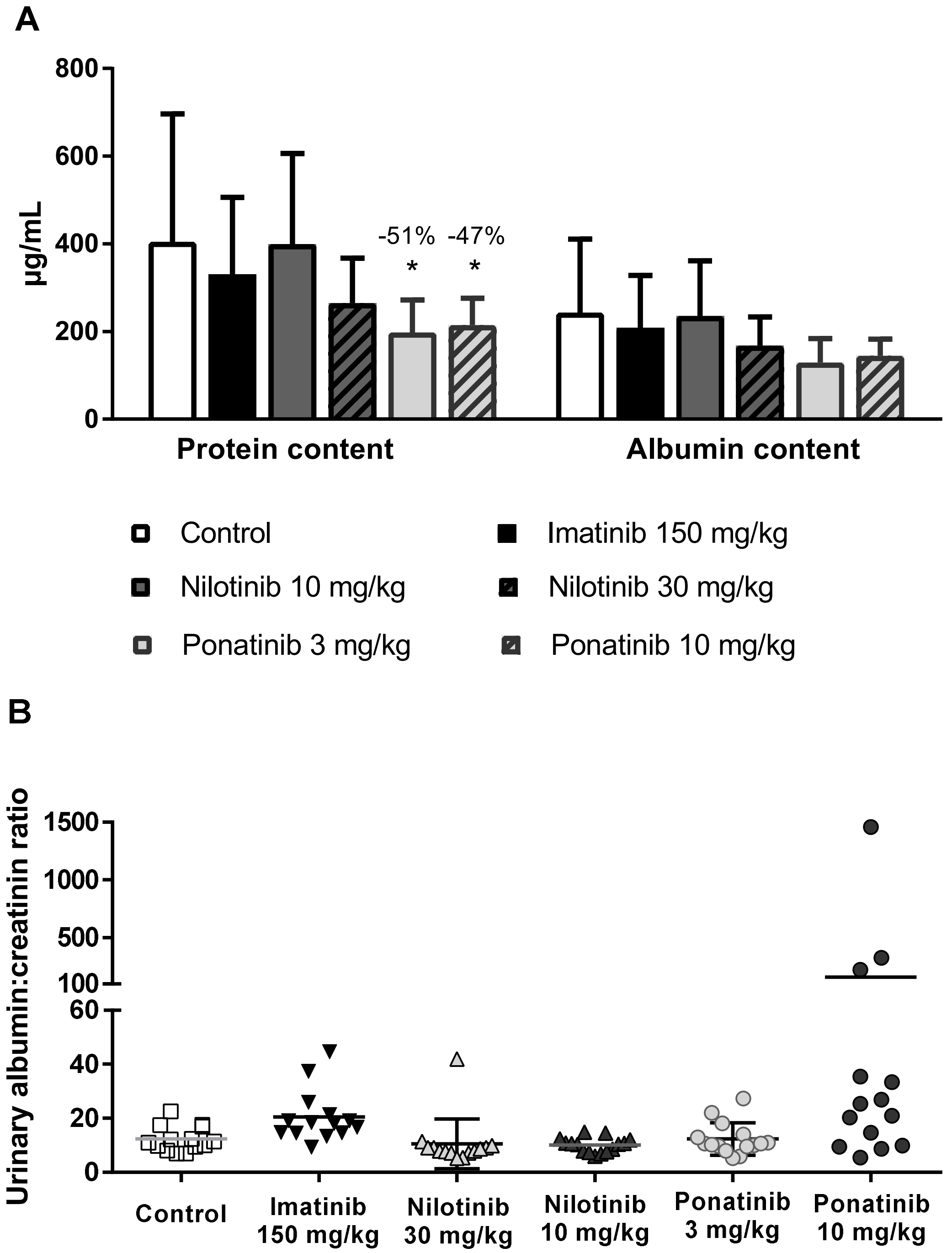

Supplemental figure 1. Ponatinib decreases albumin content in BAL fluid and urinary albumin:creatinin ratio is not affected by TKI treatment. Broncho-alveolar lavage (BAL) fluid was collected directly after sacrifice at week 16 **(A)**. Urine was collected at week 13 and the albumin:creatinin ratio was assessed **(B)**. * P<0.05. Data are presented as means ± SD (n = 12-15 per group).

A

	Number of differentially expressed genes versus control group				
	Imatinib 150 mg/kg BID	Nilotinib 10 mg/kg QD	Nilotinib 30 mg/kg QD	Ponatinib 3 mg/kg QD	Ponatinib 10 mg/kg OQ
**Imatinib 150 mg/kg BID**	1575	224	417	213	670
**Nilotinib 10 mg/kg QD**		474	313	41	248
**Nilotinib 30 mg/kg QD**			1066	78	484
**Ponatinib 3 mg/kg QD**				504	235
**Ponatinib 10 mg/kg OQ**					1344

B

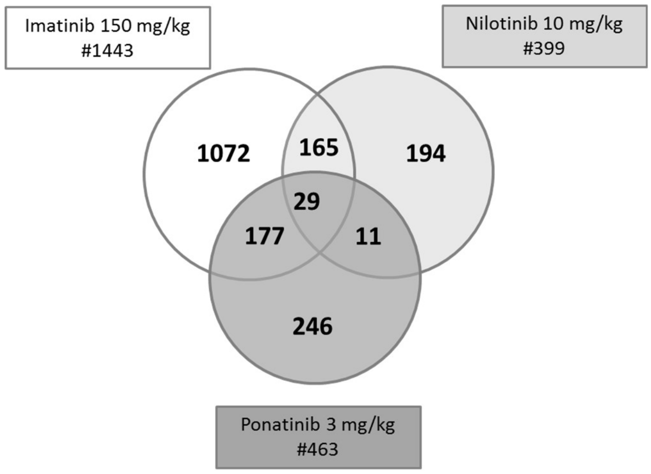

C

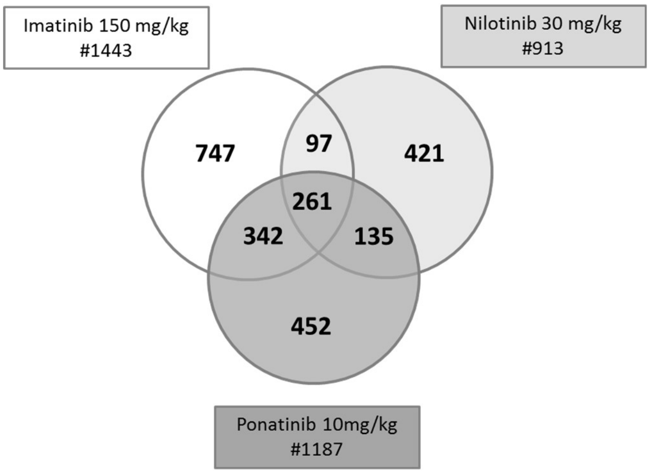

Supplemental figure 2. Overview of differentially expressed genes (DEG). The numbers in the diagonal display DEG compared to control. The numbers above the diagonal indicate the number of DEG shared between the treatment groups. Bayes p-values of <0.01 were used as cut-off **(A)**. To evaluate which biological processes are affected with the various treatments, all genes (P<0.01) were uploaded in the Ingenuity Pathway Analysis (IPA)tool to perform gene set enrichments. The number of differentially expressed genes that are annotated in IPA are indicated in Venn-diagrams **(B-C)**. This number is slightly different between A and B-C since the RNA-seq method also detects genes that are not yet annotated in the IPA database (n = 8 per group).

A

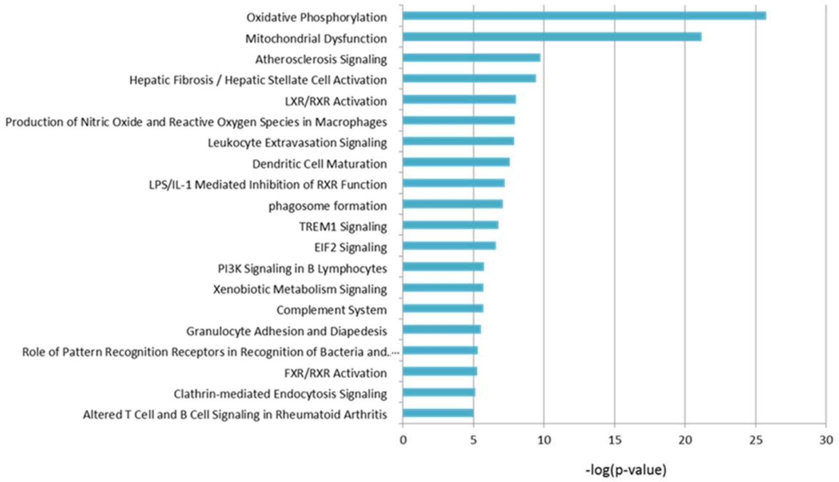

B

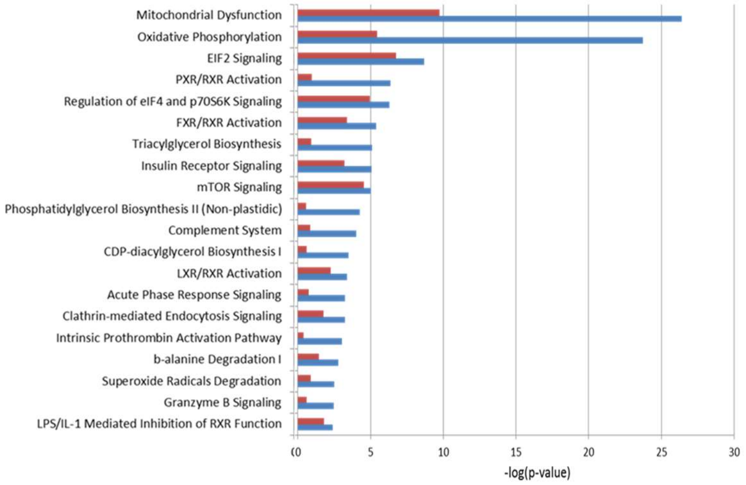

C

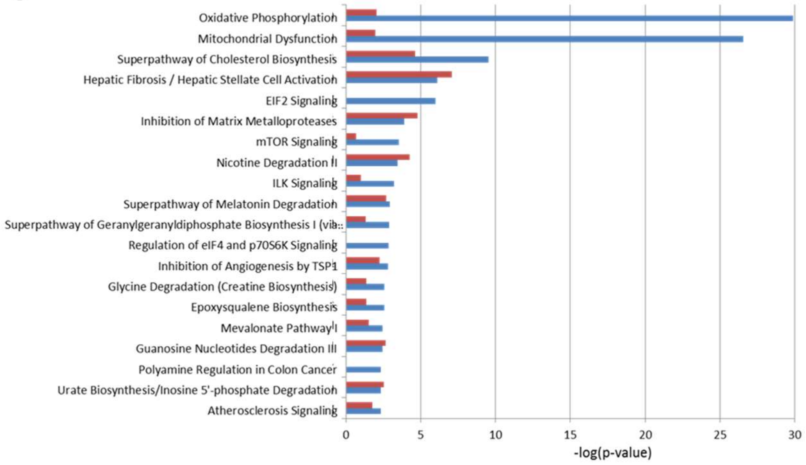

Supplemental figure 3. To identify the most relevant processes affected by TKI treatment, we calculated the canonical biological processes/pathways affected by imatinib 150 mg/kg **(A)**, nilotinib 10 mg/kg (red bars) and 30 mg/kg (blue bars) **(B)** and by ponatinib 3 mg/kg (red bars) and 10 mg/kg (blue bars) **(C)**. The relevance of each process is indicated by a p-value of overlap. The p-value of overlap is calculated based on Fisher's exact test which is set standard for overlap analysis in IPA-software. For visualization purposes the –log of the p-value of the top 20 processes are plotted on the x-axes (n = 8 per group).

A

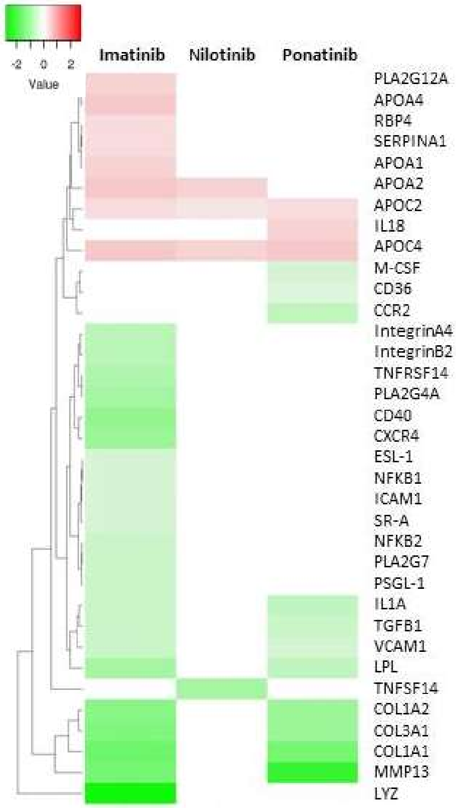

Supplemental figure 4. TKI treatment regulates many genes related to atherosclerosis signaling, with the most pronounced effect by imatinib. The heat map shows all significantly upregulated (red) and downregulated (green) genes involved in atherosclerosis signaling of mice treated with imatinib (150 mg/kg), nilotinib (30 mg/kg) or ponatinib (10 mg/kg) as compared to control mice (**A**). Molecular response of imatinib (150 mg/kg) on atherosclerosis signaling visualized by sub-pathways (**B**). P-values of <0.01 were used as cut-off (n = 8 per group).

B

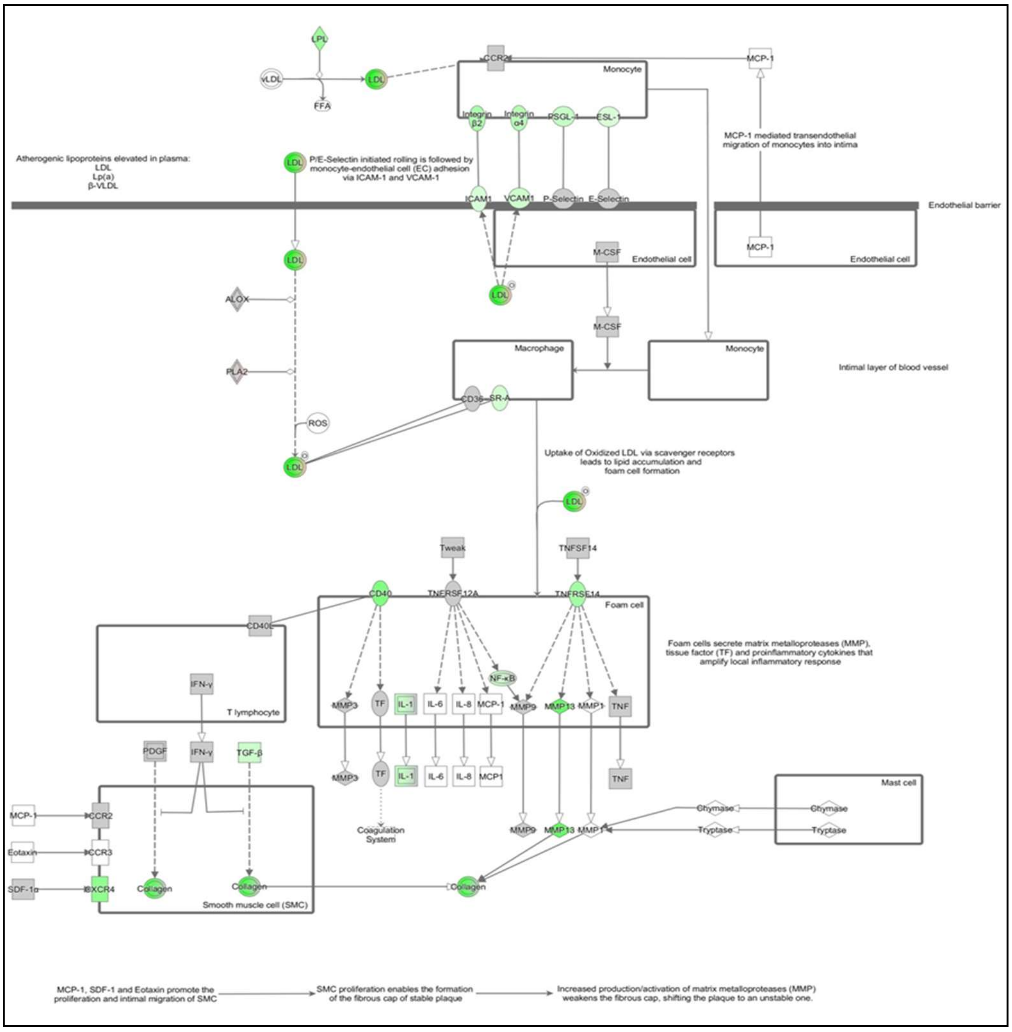

Supplement: Supplementary file 1 [file DataSheet1.pdf]
